# New Mode of Treating Hydrocele

**Published:** 1842-09

**Authors:** J. Pancoast

**Affiliations:** Prof. of Anatomy, Jefferson Medical College, Philada.


*New Mode of Treating Hydrocele.* By J. Pancoast, Prof. of Anatomy, Jefferson Medical College, Philada.—Hydrocele in children, even where the opening has been closed, that led from the tunica vaginalis to the cavity of the abdomen, is a disease of frequent occurrence. In early infancy strong discutient lotions will usually suffice for its cure. But after the second year, some more efficient means are required to produce this result. Mere evacuation of the serum with a common lancet or trochar, or a number of punctures made into the sac with a large needle, so that the fluid may escape into the cellular tissue of the scrotum, and be subsequently removed by the absorbents, are the modes of cure commonly relied on. But I have found them so uncertain in their result, success in many cases being attained only by a repetition of the process, that I have latterly adopted the following plan of treatment, which in three cases that I have tried it in has proved perfectly successful.

I puncture the swelling, in front and below its middle, with a common thumb lancet. When the serum is discharged, a little pressure causes the serous or vaginal tunic to protrude in the form of a small cyst. This I lay hold of with a pair of forceps, and draw it out, as far as it will admit. I then divide the lower half of the cyst next the skin with a pair of scissors, and traction again being made upon the pedicle, still more of the tunic may be drawn out from the upper portion of the scrotum, which is nipped partly off and treated in like manner as before. I repeat this process while any portion of the vaginal tunic can be made to protrude readily at the opening, so as to be laid hold of with the forceps. I then surround the side of the scrotum and the testicle involved with strips of adhesive plaster, after the manner of Fricke of Hamburg for the cure of hernia humoralis. By this means, the cellular tissue of the scrotum (the tunica vaginalis reflexa having been removed, to a considerable extent, with the forceps and scissors) is brought directly into contact with, and ultimately becomes adherent to that portion of the vaginal tunic which is closely attached to the fibrous coat of the testicle.

The child is allowed to run about as usual, and in a few days is perfectly well. Excepting as regards the puncture of the skin, the operation is entirely devoid of pain.

This plan of cure will, I think, be generally found applicable in children. It is certainly more speedy and certain in its results than any measure short of injection of the sac, which is not usually practised in children.

It would also, I think, be found successful in the recent hydrocele of adults, before the tunica vaginalis reflexa has become so coriaceous, or been so thickened by disease, as to prevent its being drawn out in the form of a cyst through a narrow opening. In one instance, where the puncture or palliative process had been several times tried without success, and in which I feared I might find a thickened membrane, I made the puncture through the skin with a curved bistoury, and pushing it on to the top of the sac, divided with the point of the instrument as I withdrew it, the anterior wall of the tunic, laying open the sub-cutaneous cellular tissue of the scrotum, but not cutting the skin. Subsequently, no difficulty was encountered in drawing out the tunic and removing it with the scissors.

April 20, 1842.

Since writing the above, I have operated upon another case in the same manner, in which the passage leading to the cavity of the abdomen had remained open. The cure in this instance has been slow and gradual, occupying about a month, and without the aid of a truss. The fluid re-accumulated in the tumor during the first week, but it was gradually absorbed, adhesion beginning below and proceeding upwards, until a radical cure was effected.—*Am. Med. Intelligencer.*

June 8, 1842.

